# Smad2/3‐pathway ligand trap luspatercept enhances erythroid differentiation in murine β‐thalassaemia by increasing GATA‐1 availability

**DOI:** 10.1111/jcmm.15243

**Published:** 2020-04-29

**Authors:** Pedro A. Martinez, Robert Li, Harish N. Ramanathan, Manoj Bhasin, R. Scott Pearsall, Ravindra Kumar, Rajasekhar N. V. S. Suragani

**Affiliations:** ^1^ Acceleron Pharma Cambridge MA USA; ^2^ BIDMC Beth Israel Deaconess Medical Center, Harvard Medical School Cambridge MA USA

## Abstract

In β‐thalassaemia, anaemia results from ineffective erythropoiesis characterized by inhibition of late‐stage erythroid differentiation. We earlier used luspatercept and RAP‐536 protein traps for certain Smad2/3‐pathway ligands to implicate Smad2/3‐pathway overactivation in dysregulated erythroid differentiation associated with murine β‐thalassaemia and myelodysplasia. Importantly, luspatercept alleviates anaemia and has been shown to reduce transfusion burden in patients with β‐thalassaemia or myelodysplasia. Here, we investigated the molecular mechanisms underlying luspatercept action and pSmad2/3‐mediated inhibition of erythroid differentiation. In murine erythroleukemic (MEL) cells in vitro, ligand‐mediated overactivation of the Smad2/3 pathway reduced nuclear levels of GATA‐1 (GATA‐binding factor‐1) and its transcriptional activator TIF1γ (transcription intermediary factor 1γ), increased levels of reactive oxygen species, reduced cell viability and haemoglobin levels, and inhibited erythroid differentiation. Co‐treatment with luspatercept in MEL cells partially or completely restored each of these. In β‐thalassaemic mice, RAP‐536 up‐regulated *Gata1* and its target gene signature in erythroid precursors determined by transcriptional profiling and gene set enrichment analysis, restored nuclear levels of GATA‐1 in erythroid precursors, and nuclear distribution of TIF1γ in erythroblasts. Bone marrow cells from β‐thalassaemic mice treated with luspatercept also exhibited restored nuclear availability of GATA‐1 ex vivo. Our results implicate GATA‐1, and likely TIF1γ, as key mediators of luspatercept/RAP‐536 action in alleviating ineffective erythropoiesis.

## 1 INTRODUCTION

Erythropoiesis is the process by which progenitor cells committed to the erythroid lineage develop and eventually differentiate to form red blood cells (RBC). Ineffective erythropoiesis (IE) refers to an abnormal number of erythroid progenitor cells accompanied by deficient RBC production, leading to anaemia and hypoxia. Anaemia as a result of IE is a common secondary outcome associated with diseases ranging from nutritional deficiencies ^1^ to cancers. IE occurs in disorders with deficient production of erythroblasts, such as aplastic anaemia,^2^ and in disorders with erythroid maturation defect (EMD), such as β‐thalassaemia and myelodysplastic syndromes (MDS).^3^, ^4^ IE in β‐thalassaemia is caused by mutations in the β‐globin gene leading to defective haemoglobin production,^5^ whereas IE in MDS is caused by varied mutations in haematopoietic lineage cells.^6^ Despite the diversity of factors underlying IE, the common outcome in the two aforementioned diseases is inhibition of terminal erythroid differentiation and accumulation of immature erythroid precursors in erythropoietic tissues.^3^, ^4^ Currently, the mainstay supportive treatment for patients with IE is repeated blood transfusion, which leads to progressive iron accumulation in multiple tissues and complications from iron overload despite iron chelation therapy.^7^

The TGF‐β superfamily plays a critical role in regulating haematopoiesis in normal and disease states by controlling cellular proliferation, differentiation and apoptosis.^8^, ^9^ This superfamily comprises several dozen ligands, including TGF‐β isoforms, activins, growth differentiation factors (GDFs) and bone morphogenetic proteins (BMPs), which engage promiscuously with multiple combinations of receptors to produce signals of remarkable complexity.^10^ In brief, these ligands trigger formation of heteromeric complexes between specific type I and type II transmembrane receptors, leading to phosphorylation of cytoplasmic Smad proteins, such as Smad2/3 or Smad1/5/8.^11^ Such activated Smads (pSmads) then form an oligomeric complex with Smad4 (co‐Smad) and enter the nucleus to directly alter gene transcription in combination with transcription factors, chromatin‐remodelling complexes and histone‐modifying enzymes.^8^, ^12^ Importantly, Smad signalling occurs along two main branches that often mediate opposing functional outcomes. Activins, GDF8, and GDF11 signal through Smad2 or Smad3 (Smad2/3 pathway), whereas BMPs and other GDFs typically signal through Smad1, Smad5, or Smad8 (Smad1/5/8 pathway). TGF‐β signals primarily through Smad2/3 but can also use Smad1/5/8 in certain contexts.^12^

Smad2/3 signalling has emerged as an important regulator of erythropoiesis, exerting an inhibitory influence under normal steady‐state conditions.^8^, ^9^, ^13^, ^14^ Additionally, overactivation or dysregulation of Smad2/3 signalling has been implicated in diseases characterized by impaired erythroid differentiation and IE,^9^, ^15^, ^16^, ^17^, ^18^ There is evidence for further branching of the Smad2/3 pathway because of the ability of pSmad2/3 to bind alternatively to Smad4 or TIF1γ (transcription intermediary factor 1γ), also known as TRIM33 (tripartite motif‐containing 33). In human haematopoietic/progenitor cells, where TGF‐β inhibits proliferation and stimulates erythroid differentiation, pSmad2/3‐TIF1γ complexes mediate the differentiation response while pSmad2/3‐Smad4 complexes mediate the anti‐proliferative response.^19^ Evidence has also emerged that formation of pSmad2/3‐TIF1γ complexes can be regulated by TIF1γ phosphorylation^20^ and that TIF1γ can influence Smad4 availability thorough its activity as a Smad4 ubiquitin ligase.^21^, ^22^, ^23^ Importantly, TIF1γ has been shown to stimulate expression of the master erythroid transcription factor GATA‐1 and its downstream erythroid‐signature genes in both mice and zebrafish.^24^, ^25^, ^26^ Thus, competition between TIF1γ and Smad4 for pSmad2/3 mediates TGF‐β–induced commitment of haematopoietic stem cells to the erythroid lineage, raising the possibility that a related mechanism may act at later stages to co‐ordinate erythroid differentiation.

We previously investigated the role of Smad2/3 signalling in terminal erythroid differentiation using luspatercept (ACE‐536)—a modified extracellular domain of human activin receptor type IIB (ActRIIB) attached to a human IgG1 Fc domain—and its murine analogue. These agents produce sustained elevations of haemoglobin levels in a wide variety of settings, including normal rodents, nonhuman primates and healthy volunteers^18^, ^27^ as well as in murine models of MDS or β‐thalassaemia and patients with these diseases.^17^, ^18^, ^28^ Luspatercept and RAP‐536 elevate RBC levels by a mechanism distinct from that of erythropoietin because they enhance maturation of erythroid precursors without first increasing numbers of erythroid progenitors. These agents sequester and neutralize several ligands of the Smad2/3 signalling pathway, including activin B, GDF8 and GDF11, thus leading to inhibition of Smad2/3 signalling.^18^ However, the downstream molecular effects of these agents remain to be identified.

In the present study, we investigated the molecular mechanism by which luspatercept‐mediated inhibition of Smad2/3 signalling promotes erythroid differentiation. Our results indicate that ligand‐mediated overactivation of the Smad2/3 pathway in murine erythroleukemic cells impedes their differentiation, whereas co‐treatment with luspatercept restores nuclear GATA‐1 levels together with multiple indicators of differentiation, likely through a mechanism favouring nuclear localization of TIF1γ. Corroborating evidence was obtained in β‐thalassaemic mice, in which RAP‐536 up‐regulates *Gata1* and its target gene signature in erythroid precursors as determined by transcriptomic and gene set enrichment analyses restores nuclear levels of GATA‐1 in erythroid precursors.

## 2 METHODS

### 2.1 Murine model of β‐thalassaemia

*Hbb^th1/th1^* and *Hbb^th3/+^* mice and C57BL/6 wild‐type (WT) strains were obtained from Jackson Laboratories and maintained at Acceleron Pharma. Genotyping was carried out at Transnetyx Inc, TN. All procedures used in this study were performed according to protocols that were previously approved by the Acceleron Pharma Institutional Animal Care and Use Committee.

### 2.2 Cell lines

Mouse erythroleukemic cells were obtained from DSMZ (Braunschweig, Germany—Cell line: MEL‐745A cl. DS19) and were grown in DMEM (Cat. #119905‐065, Gibco), 10% FBS (Gibco), 5% penicillin/streptomycin (Cat. #15140‐122, Gibco), 5% MEM (Cat. #11140‐050, Gibco) and 5% sodium pyruvate (Cat. #11360‐070, Gibco). MEL Cells were differentiated with 2% DMSO (Cat. #D2650, Sigma).

### 2.3 Luspatercept and RAP‐536

As previously described, luspatercept consists of a modified human ActRIIB ECD (residues 24‐131) linked to the human IgG1 Fc domain.^18^ RAP‐536, the murine analogue of luspatercept, was generated similarly as described.^18^

### 2.4 Flow cytometry

To measure ROS levels, 5‐(and 6)‐chloromethyl‐2’, 7’‐dichlorodihydrofluorescein diacetate (CM‐H_2_DCFDA; C6827, Invitrogen) was used according to the manufacturer's instructions. Positive controls for ROS were obtained by treating cells for 30 minutes with either tert‐butyl hydroperoxide solution (TBHP, C10492, Invitrogen, 400 μM) or hydrogen peroxide (Sigma, 50 μmol/L) at 37°C. Changes in GATA‐1, haemoglobin‐alpha, p‐Smad2 and p‐Smad3 expression were analysed with a mAb to GATA‐1 (Cat. #3535, Cell Signaling), haemoglobin‐alpha (Cat. #ab215919), pSmad2 (Cat. #04‐953, Millipore) and pSmad3 (Cat. #52903, Abcam). Donkey anti‐rabbit AF488 (A21206, Thermo Fisher) was used as a secondary antibody. Cells were incubated with the respective conjugated antibodies for 20 minutes in FBS Stain Buffer (Cat. #554656, BD Biosciences). For intracellular staining, cells were permeabilized with BD Perm/Wash Buffer (Cat. #51‐2091KZ) for 20 minutes. Fc Blocker (Cat. #553141, BD Biosciences) was used. Cells were then washed and analysed on a flow cytometer.

The various sub‐populations of erythroid precursors found in the splenocytes were stained with CD71‐FITC (transferrin receptor, Cat. #553266, BD Biosciences) and TER119‐PE (glycophorin‐A–associated protein, Cat. #553673, BD Biosciences) antibodies. Cells were then sorted on MoFlo, and FACS Aria sorters at the Tufts Laser Cytometry Core Facility (Tufts University School of Medicine, Boston, MA).

### 2.5 Immunofluorescence

Cells were fixed in 4% PFA (Cat. #BM‐155, Boston BioProducts) for 15 minutes, followed by permeabilization, and blocking in 5% BSA and 10% secondary host serum for 60 minutes. GATA‐1 (Cat. #3535, Cell Signaling) was used at a 1:100 dilution, and a secondary donkey anti‐rabbit AF488 (A21206, Thermo Fisher) ab was used at a 1:200 dilution. Cells were left incubating in primary antibody overnight at 4°C. The next day, cells were washed with PBS and incubated in secondary for one hour. Coverslips were mounted with anti‐fade DAPI (Cat. #P36962, Invitrogen) and analysed on a Zeiss confocal LSM 880 (Beth Israel Deaconess Medical Center, Boston). Imaris image analysis software was used to quantitate the immunofluorescence data.

### 2.6 Western blot analysis

Cells were incubated overnight at 4°C with antibodies against: GATA‐1 (cat. #3535, Cell Signaling), pSmad2 (cat. #04‐953, Millipore), pSmad3 (cat. #52903, Abcam), tSmad3 (cat.#40854, Abcam), PU.1 (cat. #88082, Abcam), TIF1 (cat# 33475, Abcam) and TIF1γ (Cat# sc‐101179, santacrutz) all used at 1:1000 dilution and left overnight at 4°C. Detection was via a peroxidase‐conjugated anti‐rabbit antibody (cat. #111‐035‐003, Jackson Laboratories) or peroxidase‐conjugated anti‐mouse antibody (cat. # 115‐035‐003, Jackson Laboratories), incubated at a 1:20 000 dilution for 1 hour at RT. GAPDH (cat. #5174S, Cell Signaling) was used as a total cell extract control and Lamin B1 (cat. #13435S, Cell Signaling) as loading controls for nuclear extracts. Membranes were then incubated in ECL (cat. #80196, Pierce) for 5 minutes, washed and developed. Ligands were used at the following concentrations: GDF11 (100 ng/mL), activin B (10 ng/mL), GDF8 (100 ng/mL) and activin A (10 ng/mL) alone or in combination with luspatercept (1 μg/mL). HT‐1080 cell extracts treated with hTGFβ3 (cat# 12052S, Cell Signaling) serve as the positive control for pSmad2/3 in respective figures.

### 2.7 Real‐time quantitative PCR assay

Cells were placed into RNAprotect Cell Reagent (Cat. #76526, Qiagen), and RNA was isolated by using an Aurum total RNA mini kit (Cat. #732‐6820, Bio‐Rad). cDNA was synthesized by using an iScript cDNA kit (Cat. #172‐5037, Bio‐Rad). PrimePCR Primers (Bio‐Rad) and housekeeping control genes were used for data acquisition. Data analysis was done with the CFX Manager Software (Bio‐Rad).

### 2.8 Transcriptome analysis

After cell sorting, sorted populations were used for RNA extraction with an RNeasy mini kit (Cat. #74106, Qiagen) as per the manufacturer's protocol. RNA quality was analysed via a BioAnalyzer and checked for RNA degradation prior to running RNA sequencing using Illumina paired‐end sequencing approach. Sequencing libraries were generated from double‐stranded cDNA using the Illumina TruSeq kit according to the manufacturer's protocol. Library quality control was checked using the Agilent DNA High Sensitivity Chip and qRT‐PCR. High‐quality libraries were sequenced on an Illumina HiSeq 2500 platform. To achieve comprehensive coverage for each sample, we generated 40‐50 million paired‐end reads.

### 2.9 Statistical analyses

GraphPad Prism was used for one‐way ANOVA, Tukey's, and non‐paired Student's t test when appropriate.

### 2.10 Data analysis

The raw sequencing data were processed to remove any adaptor, PCR primers and low‐quality transcripts using FASTQC and Trimomatic software. These high‐quality, clean reads were aligned against mouse genome (10 mm) using tophat2 and bowtie2 packages (http://tophat.cbcb.umd.edu/). Gene expression measurement was performed from aligned reads by counting the unique mapped reads. The read count‐based gene expression data were normalized based on library complexity and gene variation using Bioconductor EdgeR package. The normalized count data were compared among groups using a negative binomial model to identify differentially expressed genes. The differentially expressed genes were identified based on multiple tests corrected *P* value and fold change. Genes were considered significantly differentially expressed if the adjusted *P*‐value was < .05 and absolute fold change > 1.5. Unsupervised analysis was performed using principal component analysis (PCA), which projects multivariate data objects onto a lower dimensional space while retaining as much of the original variance as possible.

### 2.11 Gene set enrichment analysis to understand molecular mechanism of RAP‐536 treatment

As a complementary approach, we also performed analysis on normalized RNASEQ data using gene set enrichment analysis (GSEA) to determine whether a priori defined sets of genes showed statistically significant, concordant differences between transcriptome profile of control and RAP‐536 treated samples. GSEA can be more powerful than single‐gene methods for understanding effects of RAP‐536 on pathways and biological gene set levels resulting in repairing defects in erythroid maturation in thalassaemia. GSEA was performed using the GSEA‐R, a Bioconductor implementation of GSEA.^29^ GSEA was performed on pre‐ranked gene lists based on log fold change representing the effect of RAP‐536. We have performed the enrichment analysis using the canonical pathways, biological processes and transcription factor targets gene sets derived from MSigDB2.0.^29^, ^30^ The gene sets with a nominal *P*‐value (NPV) less than 5% after 1000 random permutations were considered significantly altered.

### 2.12 Comparison of RAP‐536 treatment signature with GATA‐1–regulated genes

Genome‐level GSEA demonstrated that RAP‐536 treatment significantly alerted GATA‐1–regulated genes. To further understand the role of GATA‐1 in RAP‐536 treatment, we performed a comparison of RAP‐536 and GATA‐1 transcriptome profiles using individual gene‐based, and gene set enrichment–based approaches. GATA‐1–mediated gene activation and repression signatures were obtained from the previous report.^31^ GSEA was run with 1000 permutations, and a classic statistic. Normalized enrichment score (NES) and nominal *P* values were measured to determine the significance of enrichment.

### 2.13 Pathway enrichment analysis

Pathway enrichment analysis was performed to identify pathways that are regulated/co‐regulated by luspatercept treatment and GATA‐1 transcription factor. Ingenuity Pathway Analysis (IPA 8.0, Qiagen) was used to identify the pathways that are significantly affected by RAP‐536 and GATA‐1 co‐regulated genes. The knowledge base of this software consists of functions, pathways and network models derived by systematically exploring the peer‐reviewed scientific literature. A detailed description of IPA analysis is available at the Ingenuity Systems’ web site (http//www.ingenuity.com). A *P*‐value is calculated for each pathway according to the fit of users’ data to the IPA database using one‐tailed Fisher exact test. The pathways with *P*‐values < .05 were considered significantly affected. The genes from enriched pathways and GSEA gene set were merged into functional network modules on the interaction information obtain from public databases such as MIPS, DIPS and MsigDB 2.0. The network was developed and visualized using Cytoscape: An Open Source Platform for Complex Network Analysis and Visualization.^32^

## 3 RESULTS

### 3.1 Luspatercept inhibits Smad2/3 phosphorylation in murine erythroleukemic cells

**Figure 1:**
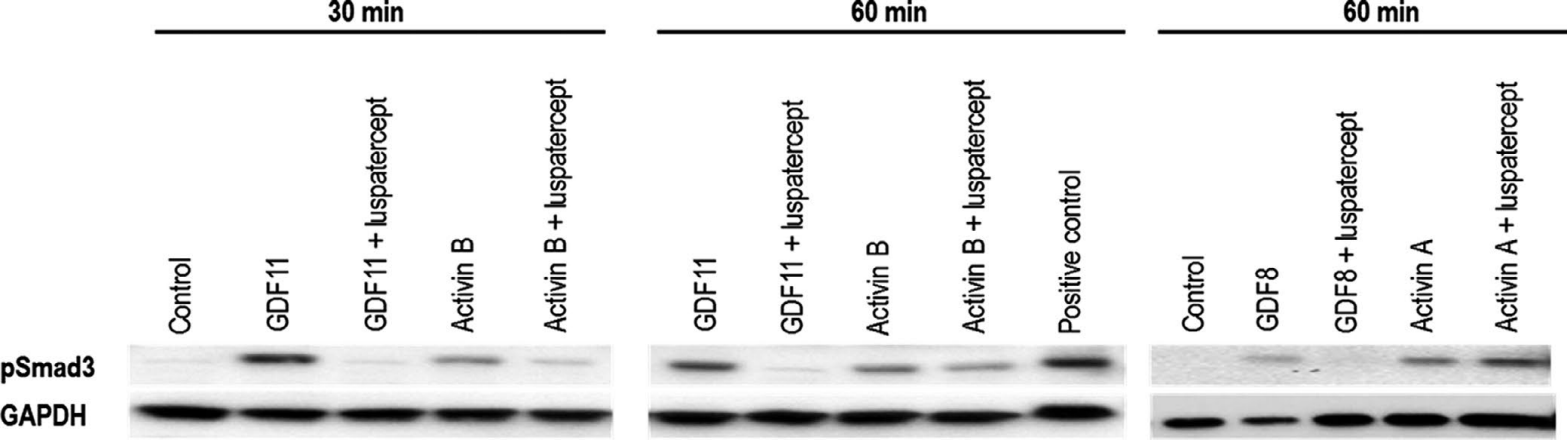
Luspatercept inhibits Smad2/3 activation by activin B, GDF8 and GDF11 in mouse erythroleukemic cells. Western blot analysis showing effect of GDF11 (100 ng/mL), activin B (10 ng/mL), GDF8 (100 ng/mL) and activin A (10 ng/mL) alone or in combination with luspatercept (1 μg/mL) on pSmad2/3 levels in MEL cells pretreated with 2% DMSO (control) to induce differentiation. Control lane represents untreated MEL cells, and HT‐1080 cell extracts treated with hTGFβ3 serve as the positive control

To investigate the mechanism by which Smad2/3 signalling regulates erythroid differentiation, we used murine erythroleukemic (MEL) cells, a well‐characterized model system that undergoes erythroid differentiation in vitro after exposure to DMSO.^33^ We first tested the ability of TGF‐β superfamily ligands to induce phosphorylation of Smad2/3 in these cells. Control lane represents untreated MEL cells, and HT‐1080 cell extracts treated with hTGFβ3 serve as the positive control. As expected, GDF11 and activin B increased levels of pSmad3 in MEL cells at both 30 and 60 minutes after treatment (Figure 1). Total Smad3 levels remained unchanged by various treatments (Figure S1A). Co‐treatment with luspatercept blocked pSmad3 induction by GDF11 at both time points and also reduced pSmad3 induction by activin B (Figure 1). As expected, co‐treatment with luspatercept also blocked pSmad3 induction by GDF8 at 60 minutes after treatment but did not inhibit pSmad3 induction by activin A (Figure 1), consistent with the previously demonstrated inability of luspatercept to bind activin A in a cell‐free system or inhibit activin A–mediated signalling in a cellular system.^18^

### 3.2 Luspatercept reverses pSmad2/3‐mediated reduction in nuclear TIF1γ levels in differentiating MEL cells

**Figure 2:**
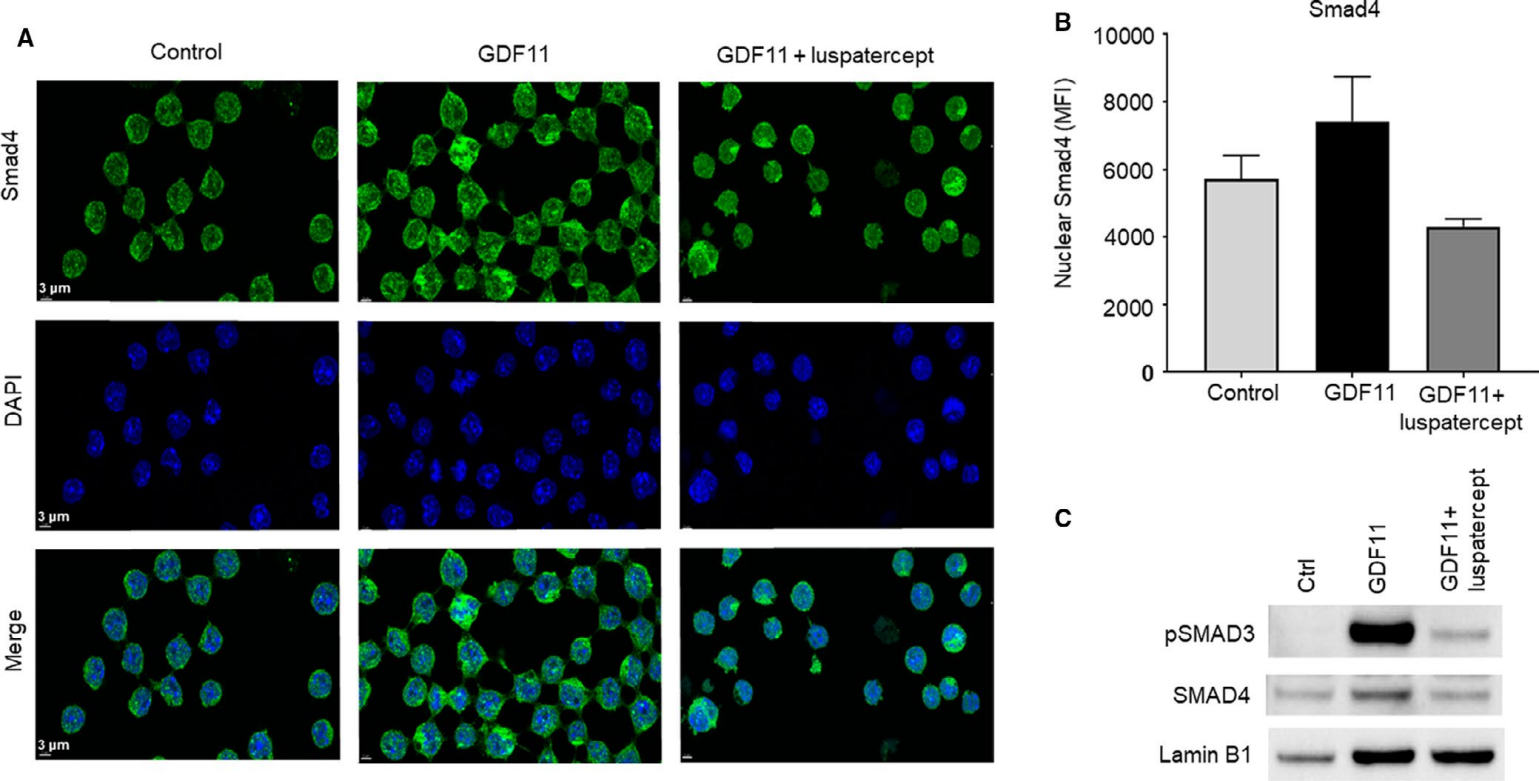
Smad2/3‐pathway overactivation increases, and luspatercept co‐treatment reduces, nuclear localization of Smad4 in MEL cells. A, Immunofluorescence microscopy showing effect of GDF11 (100 ng/mL, 24 h) alone or in combination with luspatercept (1 µg/mL) on cellular distribution of Smad4 in DMSO‐pretreated MEL cells (control). Images depict Smad4 (green/AF488) with DAPI‐labelled nuclei (blue). Scale bar, 3 µm. B, Smad4 levels determined by mean fluorescence intensity. Data are means ± SEM, (n = 3 images per group). *P*‐value .0521. C, Western blot analysis showing effect of GDF11 (100 ng/mL) alone or in combination with luspatercept (1 μg/mL) on nuclear levels of pSmad3 and Smad4 in MEL cells pretreated with 2% DMSO (control) to induce differentiation. LaminB1 served as nuclear protein loading control

Next, we studied the effect of luspatercept on GDF11‐mediated changes in the subcellular localization of phosphorylated‐Smad3, Smad4 and TIF1γ in MEL cells. When Smad2/3 is phosphorylated in the cytoplasm, it binds Smad4 and this complex translocates to the nucleus where it regulates transcription of target genes.^34^ By western blotting analysis, we found increased nuclear localization of pSmad3 in GDF11 treated MEL cells various time points upto 24 hours compared to control treated cells. Lamin B1 was used as a protein loading control for nuclear extracts (Figure S1B). Treatment of luspatercept in combination with GDF11 inhibited the nuclear localization of pSmad3 levels in a time‐dependent manner (Figure S1B). Consistent with the detection of pSmad3 in nuclear extracts, immunofluorescence microscopic analysis of MEL cells treated with GDF11 alone for 24 hours increased subcellular localization of Smad4 as determined by mean fluorescence intensity using DAPI counterstain to identify cell nuclei (Figure 2A). Nuclear Smad4 levels trended higher with GDF11 treatment alone compared to DMSO control and lower with co‐treatment of GDF11 and luspatercept, narrowly missing statistical significance (Figure 2B). Similarly, we also found increased levels of Smad4 by Western blot analysis in nuclear extracts following GDF11 treatment compared to control, and luspatercept treatment reduced pSmad3 and Smad4 levels in the nucleus (Figure 2C).

**Figure 3:**
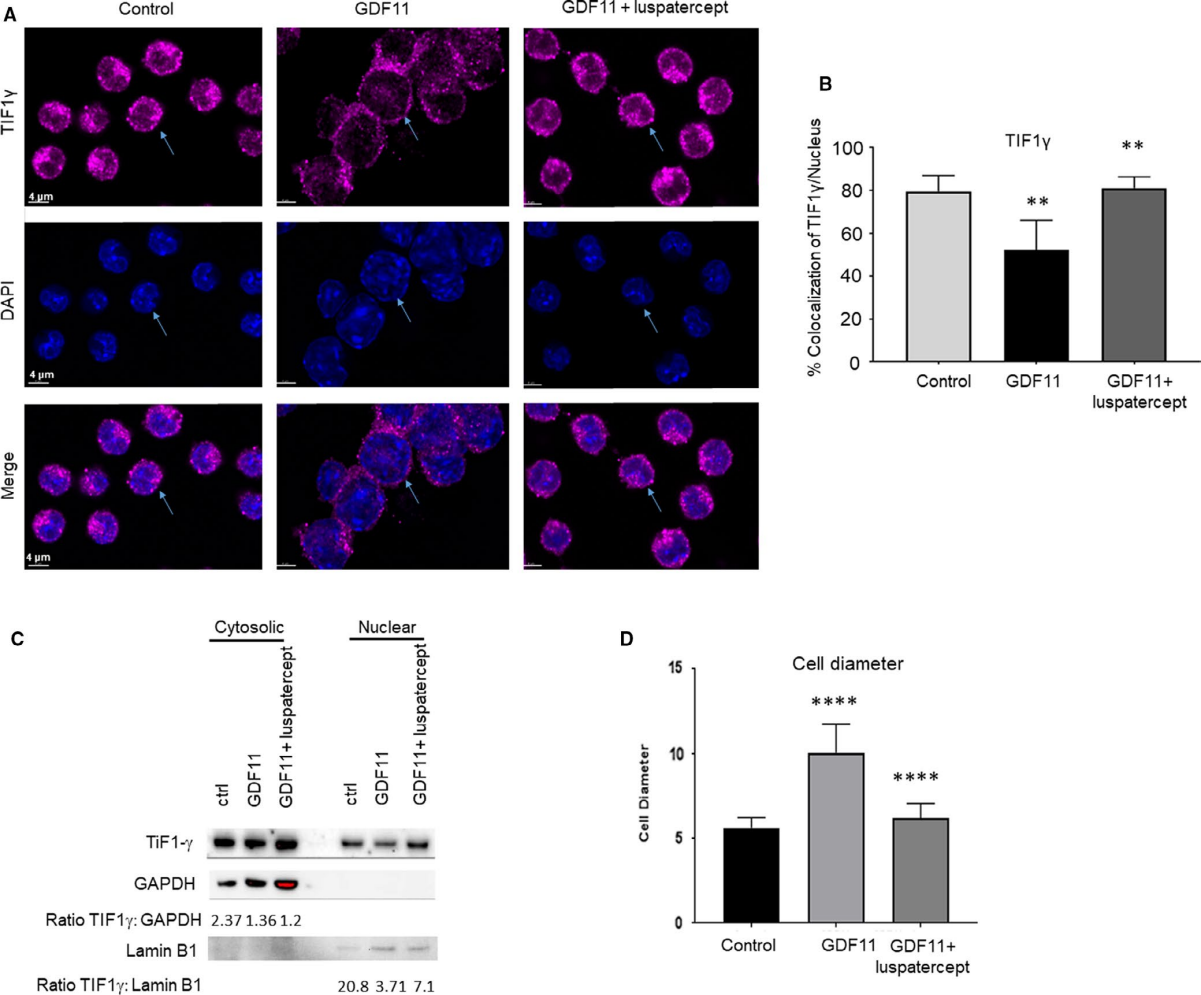
Smad2/3‐pathway overactivation reduces, and luspatercept co‐treatment promotes, nuclear localization of TIF1γ in MEL cells. A, Immunofluorescence microscopy showing effect of GDF11 (100 ng/mL, 24 h) alone or in combination with luspatercept (1 µg/mL) on TIF1γ levels in DMSO‐pretreated MEL cells (control). Representative images depict TIF1γ (magenta/AF647). TIF1γ levels were lower overall and localized to a lesser degree in the nucleus under GDF11‐treated conditions compared with control and luspatercept co‐treatment. Scale bar, 4 µm. B, Percentage of cells with nuclear TIF1γ localization. Data are means ± SEM (n = 3 images per group), *P < .05 vs. DMSO or co‐treatment. C, Western blot analysis showing effect of GDF11 (100 ng/mL) alone or in combination with luspatercept (1 μg/mL) on TIF1γ protein expression in cytosolic and nuclear fractions of MEL cells pretreated with 2% DMSO (control) to induce differentiation. GAPDH served as loading control for cytosolic extracts, and LaminB1 is used as nuclear protein loading control for nuclear extracts. D, Cell diameter measurement from panel A. Data are means ± SEM (n = 10 randomly selected cells per group), ****P < .0001 vs. control or co‐treatment. Note larger size of GDF11‐treated cells compared to control (indicated by arrowheads)

We then performed a similar experiment in MEL cells to determine effects of GDF11, with and without luspatercept, on the subcellular localization of TIF1γ. Previous studies have indicated that nuclear pSmad2/3‐TIF1γ complexes promote commitment to erythroid differentiation in haematopoietic progenitor cells,^19^ and we hypothesized that a related mechanism may influence later‐stage differentiation of erythroid precursors. Analysis of MEL cells by immunofluorescence microscopy revealed that, under control conditions (DMSO only), TIF1γ was detectable in the nucleus in approximately 80% of such cells (Figure 3A,B). GDF11 treatment caused a significant reduction in the percentage of cells with nuclear localization of TIF1γ (Figure 3A,B). Photomicrograph analysis indicated that co‐treatment with luspatercept increased the TIF1γ (magenta) immunofluorescence in the nucleus compared to that with GDF11 alone (Figure 3A,B). The findings were also corroborated by Western blot analysis in cytosolic and nuclear extracts (Figure 3C). Lamin B1 and GAPDH served as protein loading controls for nuclear and cytoplasmic extracts, respectively (Figure 3C). GDF11 treatment for 24 hours reduced TIF1γ protein expression marginally in the cytoplasmic extracts but significantly in the nuclear extracts compared to control treatment (Figure 3C). Co‐treatment of luspatercept with GDF11 increased the TIF1γ protein levels in the nuclear extracts but not in cytosolic extracts (Figure 3C). Together, these results in MEL cells indicate that the subcellular distributions of Smad4 and TIF1γ change in opposite directions as a function of Smad2/3 activation, consistent with competitive sharing between Smad4 and TIF1γ for binding to pSmad2/3 in haematopoietic stem cells as described previously.^19^

### 3.3 Smad2/3‐pathway overactivation in MEL cells reduces viability and inhibits erythroid differentiation

**Figure 4:**
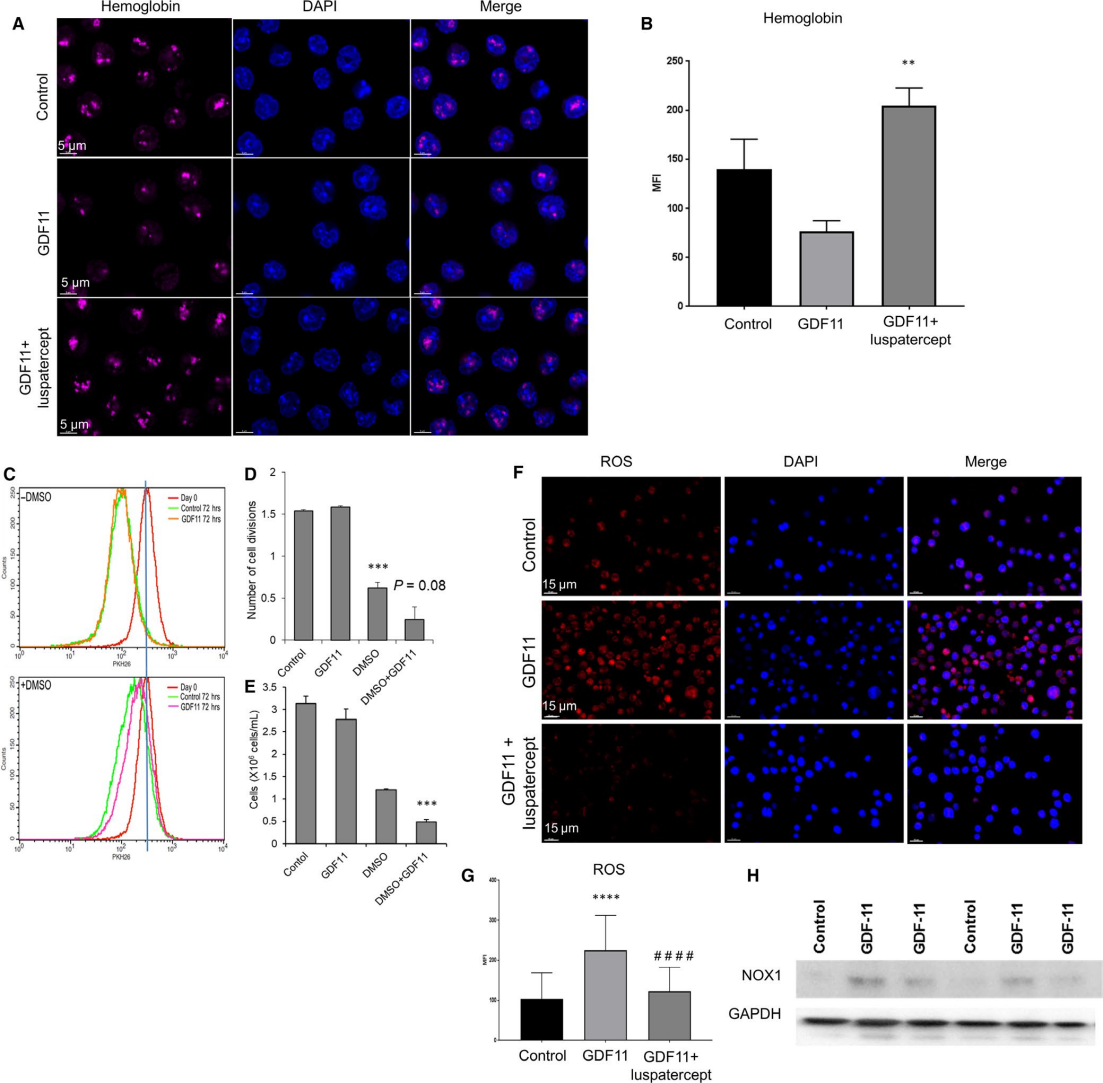
Smad2/3‐pathway overactivation reduces MEL cellular proliferation and haemoglobin levels, increases ROS and prevents nuclear condensation typical of erythroid differentiation. A, Immunofluorescence microscopic images of MEL cells showing haemoglobin‐α (magenta/AF647) as a way of measuring haemoglobin, DAPI, and a merge of the two signals. Scale bar, 5 µm. B, Haemoglobin‐α levels determined by mean fluorescence intensity. Data are means ± SEM (n = 3 images per group), GDF11‐luspatercept vs. control **P < .05. C, Cell‐division histograms (based on PKH26 staining) at baseline and 72 h as a function of treatment with GDF11 (100 ng/mL) or DMSO + GDF11. D, Number of cell divisions at 72 h. Data are means ± SEM, ***P < .001 vs. control. E, Cell density at 72 h. Data are means ± SEM, ***P < .001 vs. DMSO. F, Reactive oxygen species staining visualized via immunofluorescence in MEL cells pretreated with DMSO treated additionally with GDF11 (100 ng/mL) or combined GDF11 (100 ng/mL) and luspatercept (1 µg/mL) for 24 h. Scale bar, 50 µm. G, Bar graph representing relative fluorescence intensity for ROS levels from panel D. Cells with positive fluorescence were considered for quantitative analysis. ****P < .0001 control treatment vs GDF11, ^####^ *P* < .0001 GDF11 vs GDF11 + luspatercept treatment. H, NOX1 protein expression by Western blot analysis of DMSO‐pretreated MEL cells (control) treated additionally with GDF11 (100 ng/mL) or combined GDF11 (100 ng/mL) and luspatercept (1 µg/mL) for 24 h

Previous studies have indicated that increased nuclear pSmad2/3‐Smad4 and/or TIF1γ deficiency inhibits erythroid differentiation.^19^ We also examined effects of GDF11‐induced Smad2/3‐pathway overactivation on erythroid differentiation in MEL cells by assessing cell size and cellular haemoglobin levels. By visual inspection, DMSO‐induced control cells were relatively uniform in size, whereas those treated with GDF11 for 24 hours displayed a wider range of cell sizes including many larger than typically observed under control conditions (Figure 3A, indicated by arrow heads; and Supplementary Figure 2A). Measurement of 10‐15 randomly chosen cells from each treatment condition confirmed that GDF11 significantly increased mean cell diameter compared to control when quantified (Figure 3D; Figure S2A,B), thus strongly suggesting the presence of more immature progenitors under GDF11‐treated conditions. Importantly, co‐treatment with luspatercept blocked this increase in mean cell size (Figure 3A,D; Figure S2A,B). We then used immunofluorescence microscopy to assess pan cellular levels of haemoglobin (using anti‐haemoglobin‐α antibody) as a measure of cellular differentiation (Figure S2C). Consistent with its increase in cell size, GDF11 treatment for 24 hours reduced cellular haemoglobin levels compared to control conditions (Figure 4A,B), thus providing additional evidence that erythroid differentiation in MEL cells was inhibited by GDF11. Importantly, co‐treatment with luspatercept increased cellular haemoglobin levels significantly compared to either group (Figure 4A,B), thus indicating enhanced erythroid differentiation even compared to control conditions. Together, these results establish that erythroid differentiation of MEL cells responds to overactivation and inhibition of Smad2/3 signalling as expected from previous studies in other models.^18^

Similarly, previous studies have also indicated that pSmad2/3‐Smad4 complexes mediate anti‐proliferative responses in haematopoietic progenitor cells.^19^ Therefore, we next characterized effects of Smad2/3‐pathway overactivation on proliferation in MEL cells. Based on number of cell divisions and cell density as determined by PKH26 analysis, GDF11 treatment (100 ng/mL) for 72 hours reduced proliferation and/or viability of DMSO‐induced cells but not in non‐induced cells (Figure 4C‐E). Treatment of luspatercept with GDF11 trended towards an improvement in cell viability compared to GDF11 alone albeit non‐statistically significant (Figure S3A). We then assessed whether oxidative stress and apoptosis contributed to reduced cell numbers. GDF11 treatment after 24 hours increased cellular levels of reactive oxygen species (ROS), and luspatercept co‐treatment inhibited this increase (Figure 4F,G), consistent with an effect of increased Smad2/3 signalling on cell viability. To evaluate potential sources of ROS, we determined expression of NOX1, a fundamental component of the NADPH complex in erythrocytes ^35^ and a primary candidate for production of ROS. It remains to be confirmed that the increased levels of reactive oxygen species shown here with GDF11 treatment are causally related to reduced cell viability; however, our correlative finding is consistent with a previous report.^36^

### 3.4 Luspatercept reverses Smad2/3‐mediated reduction in nuclear GATA‐1 levels in differentiating MEL cells

**Figure 5:**
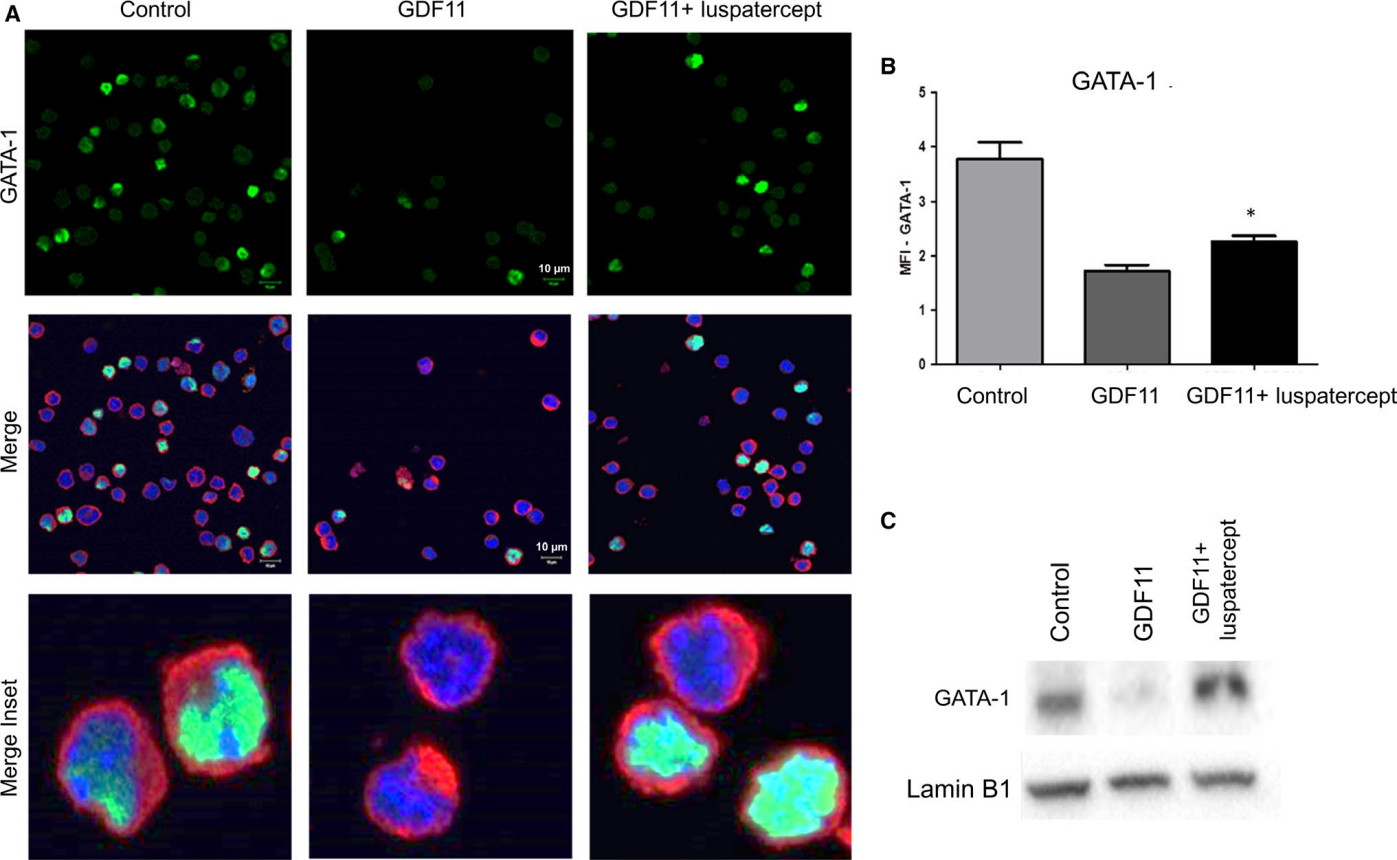
Luspatercept increases GATA‐1 nuclear availability in MEL cells. A, Immunofluorescence microscopy showing effect of GDF11 (100 ng/mL, 24 h) alone or in combination with luspatercept (1 µg/mL, 24 h) on cellular levels of GATA‐1 in DMSO‐pretreated MEL cells (control). Images depict GATA‐1 (green/AF488), HSP70 (red/AF594) and DAPI‐labelled nuclei (blue). Scale bar 10 μm. B, GATA‐1 levels determined by mean fluorescence intensity. Data are means ± SEM (n = 4 representative images per group), *P < .05 vs. GDF11. C, Western blot analysis of nuclear extracts from DMSO‐pretreated MEL cells (control) treated additionally with GDF11 (100 ng/mL) or combined GDF11 (100 ng/mL) and luspatercept (1 µg/mL). Lamin B1 was used as a nuclear loading control

Given the established role of GATA‐1 in terminal erythroid differentiation as well as links between TIF1γ and GATA‐1 expression,^26^, ^37^ we next investigated whether GDF11‐mediated activation of the Smad2/3 pathway affects GATA‐1 expression in MEL cells. As determined by visual inspection of immunofluorescence microscopy specimens, GDF11 treatment for 24 hours reduced the number of cells containing GATA‐1 compared to control conditions (Figure 5A). Co‐treatment with luspatercept increased the number of cells containing GATA‐1 compared to GDF11 alone, and examination at high magnification confirmed the nuclear localization of GATA‐1 (Figure 5A). Measurement of mean fluorescence intensity confirmed that GDF11 reduced levels of GATA‐1 per cell compared with control conditions (Figure 5B), whereas luspatercept significantly increased levels of GATA‐1 per cell compared to GDF11 alone but did not restore cellular GATA‐1 to control levels (Figure 5B). Note that this net effect of luspatercept co‐treatment on GATA‐1 protein expression reflects an increase in mean GATA‐1 levels per cell (compared to GDF11 alone, Figure 5B). To further confirm the forgoing results, we then subjected nuclear extracts from treated MEL cells to Western blot analysis. By this method, GDF11 treatment reduced nuclear GATA‐1 protein to barely detectable levels, whereas co‐treatment with luspatercept restored GATA‐1 to levels similar to control (Figure 5C). In a different experiment, we found that GATA‐1 levels trended towards lower levels in the cytosolic extracts with GDF11 treatment compared to control (Figure S3B).

We then examined in MEL cells whether changes in Smad2/3 signalling alter levels of Spi‐1/PU.1, as overexpression of this protein blocks erythroid differentiation of MEL cells through repression of GATA‐1 transcriptional activity.^38^ In undifferentiated MEL cells (without DMSO), neither acute treatment with Smad2/3‐pathway ligands nor co‐treatment with luspatercept altered Spi‐1/PU.1 expression by Western blot (Figure S3C). Similarly, in MEL cells induced to differentiate with DMSO, neither GDF11 nor co‐treatment with luspatercept altered Spi‐1/PU.1 expression, at least within 24 hours (Figure S3D). We did not investigate potential effects of longer treatment because of the confounding effect of DMSO‐induced cellular differentiation, which is accompanied by reduced expression of Spi‐1/PU.1.^37^

Collectively, our results with MEL cells indicate that GDF11‐mediated activation of the Smad2/3 pathway reduces availability of TIF1γ and GATA‐1 in the nucleus of these cells and produces traits characteristic of impaired erythroid differentiation such as larger cell size, lower haemoglobin content and increased oxidative stress. Importantly, co‐treatment with luspatercept restores nuclear availability of TIF1γ and GATA‐1 to control levels and restores traits consistent with erythroid differentiation such as smaller cell size, higher haemoglobin content, reduced oxidative stress and partly improved cell viability.

### 3.5 RAP‐536 alleviates disease comorbidities in the *Hbb^th3^* ^/+^ mouse model of β‐thalassaemia

**Figure 6:**
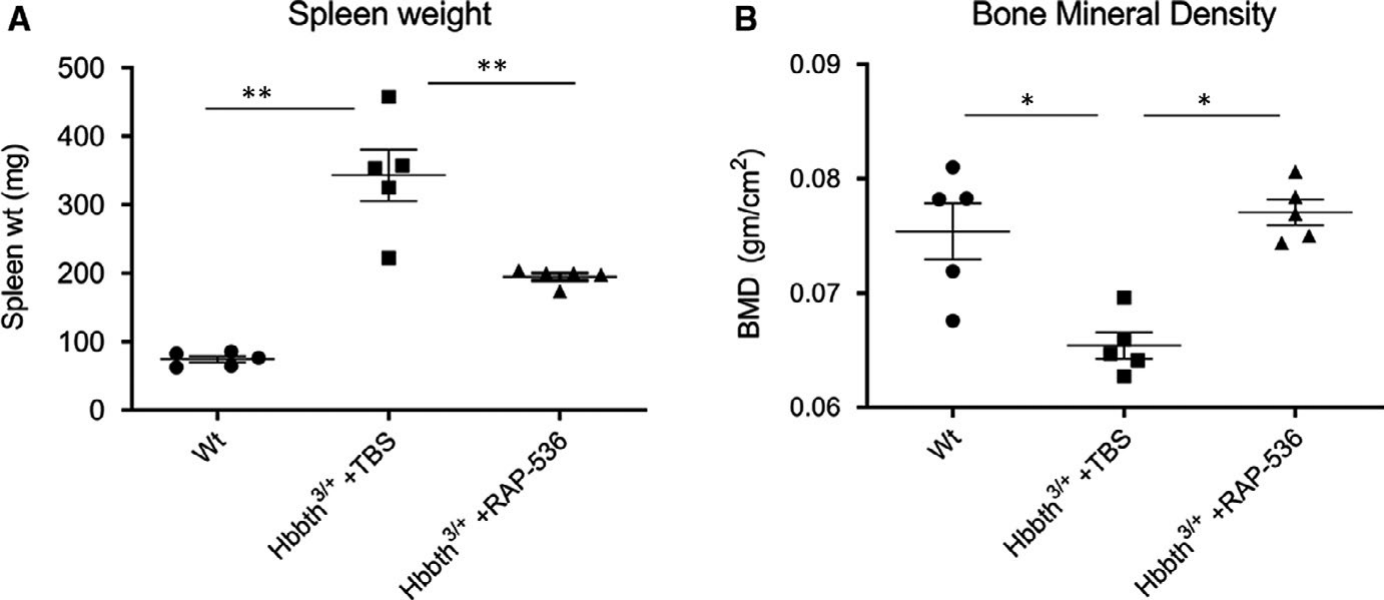
RAP‐536 alleviates disease comorbidities in *Hbb^th3^* ^/+^ β‐thalassaemic mice. A, Effect of RAP‐536 (1 mg/kg, twice weekly for 2 months) on spleen weight. Shown are means ± SEM (n = 5 mice per group). ^**^ *P* < .01 by one‐way ANOVA with post hoc Tukey HSD test. B, Effect of RAP‐536 (1 mg/kg, twice weekly for 2 months) on bone mineral density. Shown are means ± SEM (n = 5 mice per group). ^*^ *P* < .05 by one‐way ANOVA with post hoc Tukey HSD test

We have previously shown that RAP‐536 alleviates anaemia as well as comorbidities in the *Hbb^th1/th1^* mouse model of β‐thalassaemia intermedia,^17^ in which both copies of the β‐globin major gene are deleted. Here, we investigated RAP‐536 activity in the *Hbb^th3/+^* mouse model of β‐thalassaemia—in which both the β‐globin major and β‐globin minor genes have been eliminated in heterozygosity—to provide context for transcriptional profiling in this model (below). *Hbb^th3/+^* mice develop anaemia and symptoms of β‐thalassaemia intermedia, including reduced RBC parameters (Table 1), increased spleen weight (Figure 6A) and decreased bone mineral density (Figure 6A) compared to wild‐type mice. Similar to our previously reported results in the *Hbb^th1/th1^* model, RAP‐536 treatment (1 mg/kg) twice weekly for 2 months improved the phenotype in the *Hbb^th3/+^* model by increasing RBC parameters (Table 1), increasing bone mineral density (Figure 6B) and reducing splenomegaly (Figure 6A) compared to vehicle.

**TABLE 1:** RAP‐536 alleviates anaemia in *Hbb^th3/+^* mice

Group	RBC (10^6^ cells/µL)	Hb (g/dL)	Hct (%)	MCV (fL)	MCH (pg)	MCHC (g/dL)	Retic (%)	RDW_a_ (fL)
Wild‐type + Vehicle	9.64 ± 0.2	14.4 ± 3	44.1 ± 1.0	45.7 ± 0.4	15.0 ± 0.0	32.8 ± 0.2	4.2 ± 0.2^a^	29.6 ± 0.5
*Hbb^th3/+^* + Vehicle	7.6 ± 0.1***	10.1 ± 0.1***	27.9 ± 0.3***	36.6 ± 0.8***	13.3 ± 0.3***	36.3 ± 0.3***	27.9 ± 3.7^b^	34.4 ± 2.0*
*Hbb^th3/+^* + RAP‐536	10.1 ± 0.1^†††^	12.5 ± 0.2^†††^	33.4 ± 0.5^†††^	33.0 ± 0.1^†^	12.3 ± 0.1	37.4 ± 0.1	15.6 ± 0.4^†^	27.2 ± 0.4^††^

Note

Red blood cell (RBC) parameters in β‐thalassaemic (*Hbb^th3/+^*) mice treated with RAP‐536 (1 mg/kg, s.c., twice weekly) or vehicle for 2 months starting at 3‐4 months of age. Age‐matched C57BL/6 wild‐type mice served as controls. Results are expressed as mean ± SEM (n = 5 mice per group).

Hb indicates haemoglobin; Hct, haematocrit; MCH, mean cell haemoglobin, MCHC, mean corpuscular haemoglobin concentration; MCV, mean cell volume; RDW_a_, red cell distribution width area; Retic, reticulocyte.

*P < .01, ***P < .001 vs. wild‐type

^†^ *P* < .05, ^††^ *P* < .01, ^†††^ *P* < .001 vs. vehicle

^a^ N = 2.

^b^ Could not perform significance test because of insufficient N.

### 3.6 RAP‐536 up‐regulates GATA‐1 and its target gene signature in murine β‐thalassaemia

**Figure 7:**
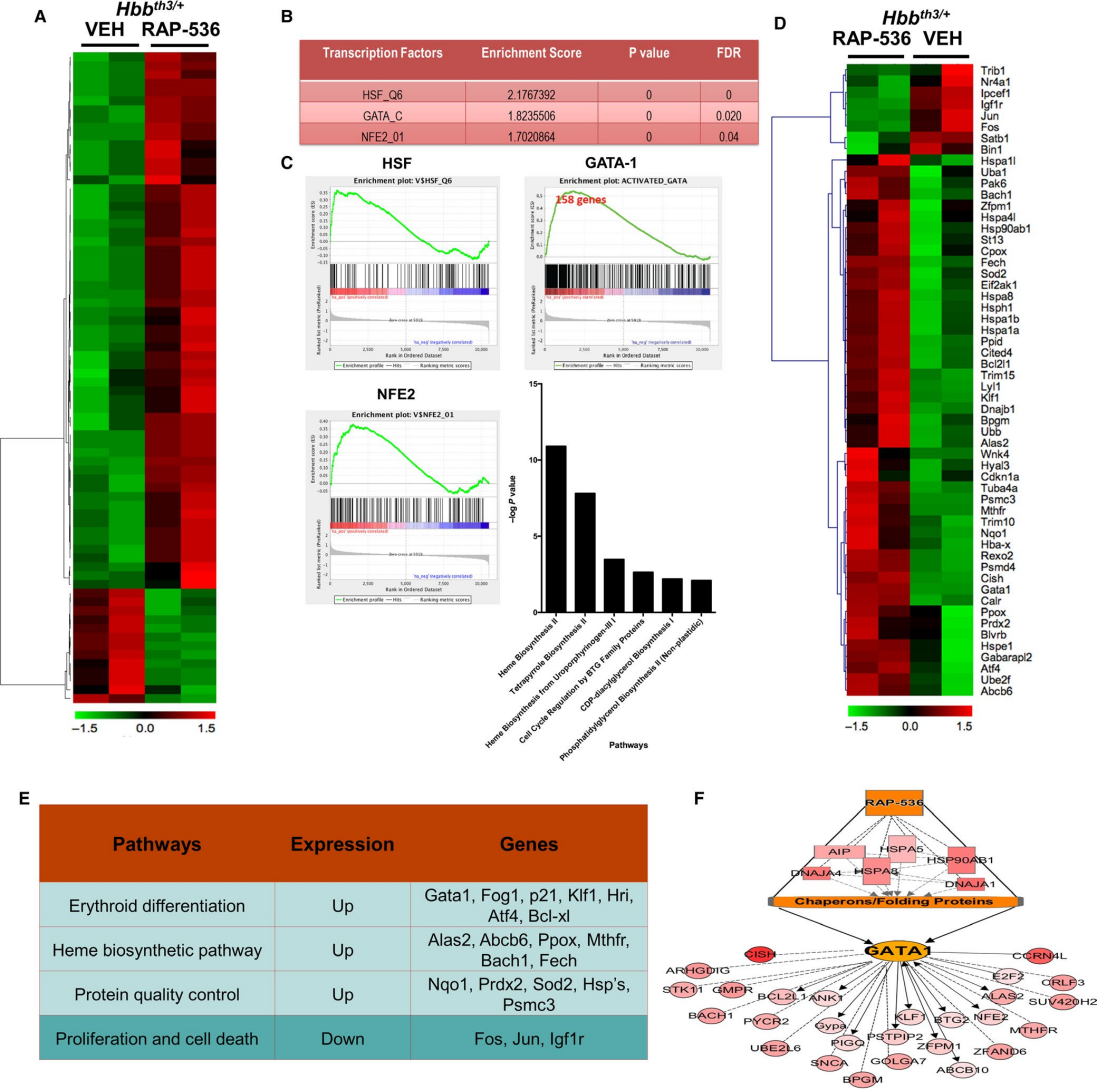
RAP‐536 up‐regulates *Gata1* and its target gene signature in erythroid precursors of *Hbb^th3/+^* β‐thalassaemic mice. A, Heat map for RNA sequencing analysis of sorted erythroid precursors from *Hbb^th3/+^* mice depicting differentially regulated genes 16 h after treatment with a single dose of RAP‐536 (30 mg/kg, i.p) or vehicle. B, Main transcription factors up‐regulated in erythroid precursors by RAP‐536 treatment. FDR—false discovery rate. C, Gene set enrichment analysis to identify those differentially regulated in erythroid precursors by RAP‐536 treatment, showing plots for GATA‐1, HSF and NFE2. Bar graph indicates pathways that were significantly activated by GATA‐1 in erythroid precursors by RAP‐536 treatment. D, Representative heat map with each column representing genes up‐regulated (red) or down‐regulated (green) in erythroid precursors of an individual mouse by treatment with RAP‐536 or vehicle (VEH). E, Pathways affected by RAP‐536 treatment and some key genes involved. F, Linkage analysis model depicting RAP‐536‐induced changes in gene expression (all up‐regulated) in erythroid precursors

To understand the primary effect of RAP‐536 on gene expression in erythroid precursors, we treated *Hbb^th3/+^* β‐thalassaemic mice with a single bolus dose of RAP‐536 (30 mg/kg). Sixteen hours after treatment, splenic basophilic erythroblasts (CD71^high^Ter119^+^Fsc^high^) were sorted by flow cytometry, and transcriptome analysis was carried out by RNA sequencing. Transcriptome analysis of β‐thalassaemic erythroblasts revealed a total of 74 genes that were differentially expressed (absolute fold change > 1.5, FDR adjusted *P* value < .05) in RAP‐536 treated samples compared to VEH treatment. Analysis depicted in the heat map (Figure 7A) shows significant up‐regulation of target genes of multiple transcriptional regulators including GATA‐1, heat shock factor and NFE2 (Figure 7B).

To identify molecular mechanisms underlying RAP‐536 activity, we performed gene set enrichment analysis (GSEA)^29^ on data from vehicle‐ and RAP‐536‐treated mice. Further GSEA of GATA‐1 activator and repressor signatures (as well as those of HSF and NFE2) against RAP‐536 treatment data revealed significant up‐regulation of 158 out of 328 activated genes (normalized enrichment score = 2.7, *P* = 0) (Figure 7C). Pathways prominently activated by GATA‐1 included haem biosynthesis and cell cycle regulation (Figure 7C, lower panel). Specific GATA‐1 target genes up‐regulated by RAP‐536 treatment include those involved in haem biosynthesis (such as *Ppox*, *Fech*, *Alas2* and *Abcb10*) and erythroid differentiation (such as *Klf1*, *Nfe2*, *Gypa*, *Bcl2l1*, *Bach1* and *Mthfr*) as shown in Figure 7D,E. No statistically significant down‐regulation of GATA‐1–repressed genes was found (data not shown). A linkage analysis model of downstream targets affected by treatment with RAP‐536 (Figure 7F) suggests that these differentially expressed genes act in concert to increase availability of GATA‐1 and thereby enhance erythroid differentiation. These results from erythroid precursors in a mouse model of β‐thalassaemia corroborate effects of luspatercept on GATA‐1 expression in GDF11‐treated MEL cells.

**Figure 8:**
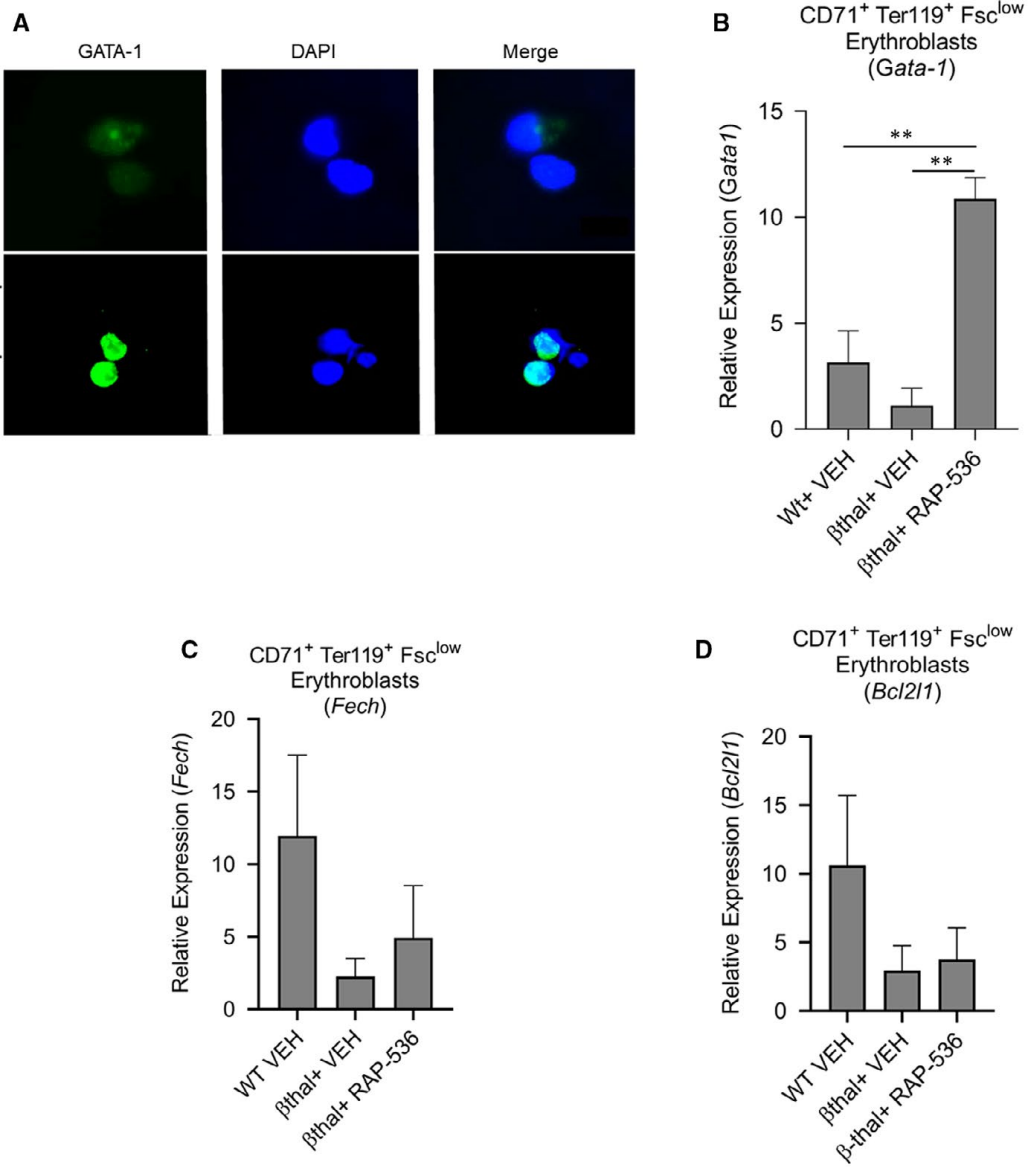
GATA‐1 levels are restored in β‐thalassaemic bone marrow cells by luspatercept in vitro and in erythroblasts of *Hbb^th1/th1^* β‐thalassaemic mice by RAP‐536 in vivo. (A) Immunofluorescence microscopy showing effect of luspatercept (1 µg/mL, 48 h) on nuclear levels of GATA‐1 in cultured bone marrow cells from *Hbb^th1/th1^* β‐thalassaemic mice. Images depict GATA‐1 (green/AF488) and DAPI‐labelled nuclei (blue). Expression of *Gata1* detected by qPCR in (B) sorted CD71^high^TER119^+^FSC^low^ splenic erythroblasts. Expression of (C) *Fech*, (D) *Bcl2l1* by qPCR in sorted splenic erythroblasts from wild‐type mice and β‐thalassaemic mice treated with a single dose of RAP‐536 (30 mg/kg) or vehicle for 16 h. Data are means ± SEM (n = 3 mice per group). ^**^ *P* < .01 vs β‐thal + RAP‐536 by one‐way ANOVA with post hoc Tukey HSD test. Data B‐F are normalized against GAPDH

As transcriptional profiling identified GATA‐1 up‐regulation as a prominent effect of RAP‐536 in *Hbb^th3/+^* β‐thalassaemic mice, we further examined effects of luspatercept or RAP‐536 on GATA‐1 levels using *Hbb^th1/th1^* β‐thalassaemic mice, a related model in which we have previously studied RAP‐536.^17^ Treatment of cultured erythroid precursors from bone marrow from β‐thalassaemic mice with luspatercept ex vivo for 48 hours produced markedly higher levels of nuclear GATA‐1 protein than vehicle treatment as determined by immunofluorescence microscopy (Figure 8A). We then used qPCR to determine mRNA levels in late basophilic and orthochromatophilic erythroblasts (CD71^med^Ter119^+^Fsc^low^) from spleens obtained 16 hours after treatment of β‐thalassaemic mice with a single dose of RAP‐536 (30 mg/kg) or vehicle. Expression of *Gata‐1* was lower, albeit not significantly, in late‐stage erythroblasts from β‐thalassaemic mice compared to erythroblasts from wild‐type mice (Figure 8B). Importantly, treatment of β‐thalassaemic mice with RAP‐536 increased *Gata‐1* expression significantly in late‐stage erythroblasts compared to either vehicle‐treated β‐thalassaemic mice or wild‐type mice (Figure 8B). In these late‐stage erythroblasts, expression of the GATA‐1 downstream target genes *Fech* (ferrochelatase, involved in haem biosynthesis) and *Bcl2l1* (Bcl‐xL, anti‐apoptotic) was non‐significantly reduced in vehicle‐treated β‐thalassaemic mice compared to wild‐type mice but was not significantly restored by RAP‐536 treatment (Figure 8C,D).

Together, these data demonstrate that overactivation of the Smad2/3 signalling pathway negatively regulates terminal erythroid differentiation in mouse models of β‐thalassaemia, partly by reducing GATA‐1 expression. RAP‐536–mediated inhibition of Smad2/3 signalling enhances erythroid maturation in this context by increasing expression and functional availability of GATA‐1.

### 3.7 RAP‐536 restores nuclear localization of TIF1γ in β‐thalassaemic erythroblasts to a focal pattern typical of wild‐type erythroblasts

**Figure 9:**
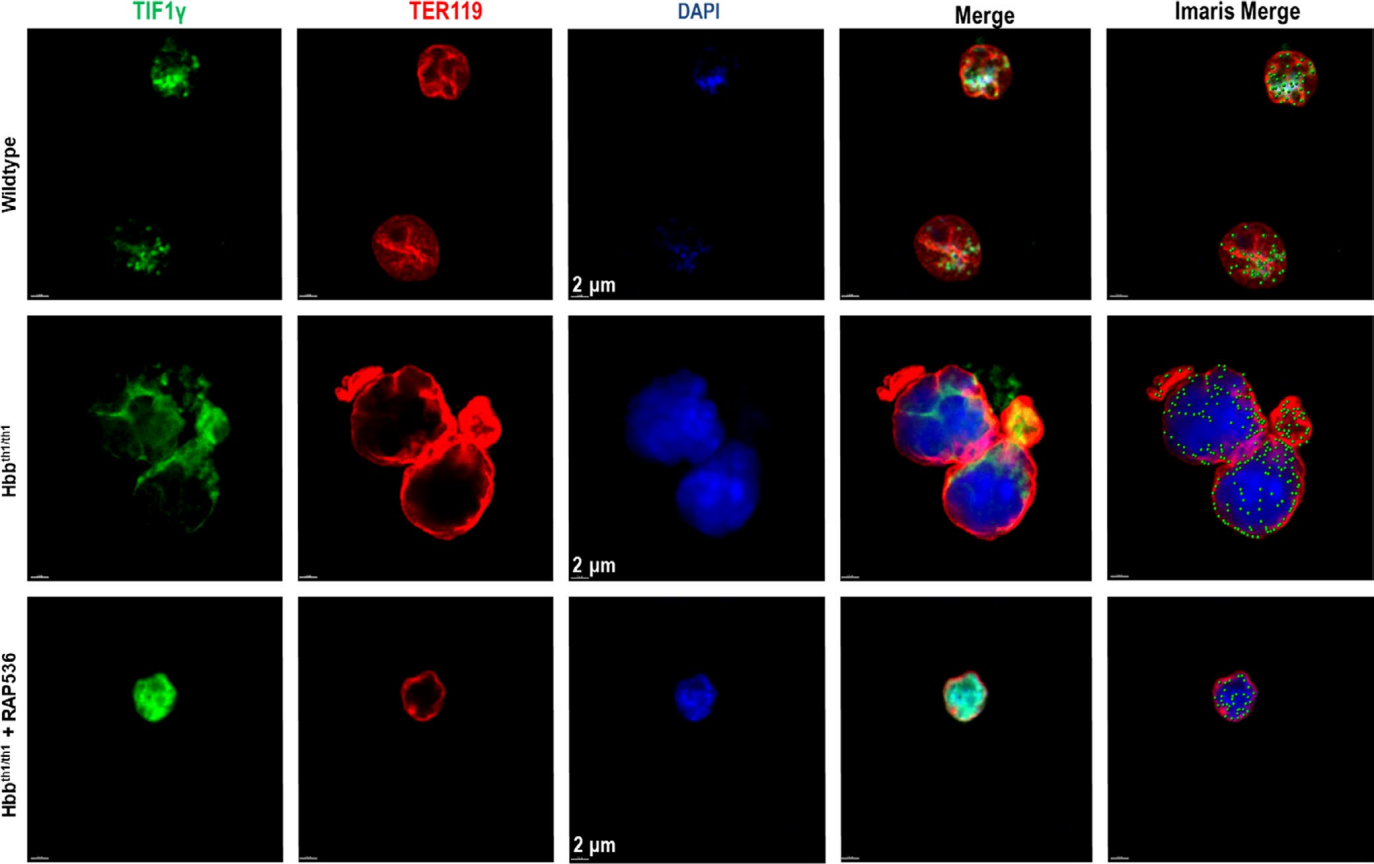
RAP‐536 treatment in vivo restores nuclear localization of TIF1γ in TER119 + bone marrow cells of *Hbb^th1/th1^* β‐thalassaemic mice to a focal pattern typical of wild‐type mice. Immunofluorescence microscopy showing the levels and subcellular distribution of TIF1γ in TER119^+^ bone marrow cells from wild‐type mice and *Hbb^th1/th1^* β‐thalassaemic mice (HOM) treated with a single dose of RAP‐536 (30 mg/kg, i.p, 16 hours) or vehicle. Images depict TIF1γ (green/AF488), TER119 (red), DAPI‐labelled nuclei (blue) and the merge of these three independent images. Scale bar, 2 µm. This merged image was further processed with Imaris software to better visualize the levels and subcellular distribution of TIF1γ by conversion of diffuse labelling to punctate labelling. As visible in the Imaris merged images, green punctate labelling representing TIF1γ is mainly distributed focally within the nuclei of erythroblasts from wild‐type mice and β‐thalassaemic mice treated with RAP‐536. By contrast, TIF1γ labelling in the noticeably larger erythroblasts from vehicle‐treated β‐thalassaemic mice is localized primarily circumferentially in the perinuclear region

Based on the ability of luspatercept to promote nuclear localization of TIF1γ in differentiating MEL cells, we lastly investigated whether RAP‐536 alters the subcellular localization of TIF1γ in erythroblasts in *Hbb^th1/th1^* β‐thalassaemic mice. We used immunofluorescence microscopy to examine TER119^+^ nucleated erythroid precursors from bone marrow of wild‐type mice or β‐thalassaemic mice treated with RAP‐536 (30 mg/kg) or vehicle for 16 hours (Figure 9). Nuclear levels of TIF1γ, as determined by DAPI counterstain, were reduced in erythroid cells from vehicle‐treated β‐thalassaemic mice compared to those from wild‐type mice or β‐thalassaemic mice treated with RAP‐536. In addition, TIF1γ was concentrated in the nuclear region of erythroid cells from wild‐type mice or β‐thalassaemic mice treated with RAP‐536, whereas TIF1γ displayed a distinct, perinuclear distribution in cells from vehicle‐treated β‐thalassaemic mice (Figure 9). It is ambivalent if the nucleated erythroid precursors shown here (Figure 9) are in the same stage of erythroid maturation, nevertheless these preliminary results do support the notion that the effects of luspatercept on IE are mediated through the subcellular distribution of TIF1γ in erythroid precursors and GATA‐1 availability.

## 4 DISCUSSION

IE occurs in multiple diseases in response to diverse triggering events. For example, β‐thalassaemia is caused by deficiency in the β‐globin subunit of haemoglobin, leading to an accumulation of unpaired α‐globin protein in developing erythroid precursors.^39^, ^40^ In contrast, MDS are caused by a spectrum of gene mutations in haematopoietic precursors.^3^ Such diverse triggering events may nonetheless promote IE by a shared underlying mechanism, as indicated by the therapeutic effectiveness of RAP‐536 in mouse models of either β‐thalassaemia or MDS as well as luspatercept in patients with either β‐thalassaemia or MDS.^17^, ^18^, ^28^, ^41^ Unlike erythropoietin, luspatercept and RAP‐536 elevate red blood cell counts by enhancing maturation of erythroid precursors without first increasing numbers of erythroid progenitors. A better understanding of the mechanisms by which luspatercept and RAP‐536 enhance erythroid differentiation could lead to novel treatment options for anaemia in these and related diseases.

We have investigated this issue in the present study using several well‐characterized model systems. MEL cells consist of erythroid progenitor cells derived from the spleens of mice infected with Friend virus and are arrested at a proerythroblast stage of development unless induced by DMSO treatment to undergo terminal differentiation into mature erythroid cells.^33^, ^42^ Here, we found that differentiating MEL cells treated with the Smad2/3‐pathway ligands activin A, activin B, GDF8 and GDF11 display elevated levels of pSmad2/3. As expected from its previously characterized ligand‐binding profile,^18^ luspatercept blocked Smad2/3 overactivation in MEL cells mediated by each of these ligands except activin A. Overactivation of the Smad2/3 pathway with GDF11 inhibited erythroid differentiation of these cells and produced phenotypic changes similar to those displayed by erythroid precursors in β‐thalassaemic mice, such as increased mean cell size, reduced viability, increased levels of ROS, and reduced levels of haemoglobin. These cellular changes in response to overactivation of the Smad2/3 pathway are consistent with evidence implicating dysregulated Smad2/3 signalling as a contributing factor in diseases with impaired erythroid differentiation and IE.^15^ Co‐treatment with luspatercept restored the erythroid differentiating phenotype in MEL cells, consistent with previously described effects of Smad2/3‐pathway inhibition in other models of IE.^15^, ^16^, ^17^, ^18^ These results support the relevance of MEL cells as a robust in vitro model of terminal erythroid differentiation for investigating luspatercept mechanism of action.

Most importantly, we find that GATA‐1 expression mediates effects of Smad2/3‐pathway activity on erythroid differentiation and that treatment with luspatercept/RAP‐536 can normalize nuclear levels of GATA‐1 in multiple models of impaired erythroid differentiation. GATA‐1 has been established as indispensable for erythroid maturation,^43^, ^44^ and its expression is known to be regulated at transcriptional, translational and post‐translational levels.^45^ Deficits in GATA‐1 expression are observed in diverse erythroid diseases, including β‐thalassaemia, MDS, congenital dyserythropoietic anaemia, Diamond‐Blackfan anaemia and myelofibrosis, thus indicating that diverse molecular defects can contribute to GATA‐1 deficiency with important consequences for erythroid differentiation and/or IE.^46^ In β‐thalassaemia and MDS, GATA‐1 nuclear availability in erythroid precursors is negatively regulated by unpaired α‐globin or dyserythropoiesis, respectively.^39^, ^47^, ^48^, ^49^ Interestingly, dysregulated Smad2/3 signalling has also been implicated in these diseases, thus raising the possibility that luspatercept may be able to mitigate IE and anaemia in these diseases. Our data showing increased nuclear availability of GATA‐1 in luspatercept‐treated erythroid precursors corroborate gene set enrichment analysis of transcriptomic data from RAP‐536‐treated β‐thalassaemic mice revealing up‐regulation of *Gata1* and its target genes involved in erythroid differentiation and haem biosynthesis. Luspatercept/RAP‐536 increased *Gata‐1* mRNA levels as well as nuclear GATA‐1 availability, and it is difficult to determine whether this treatment increased GATA‐1 expression directly or indirectly by inhibiting oxidative stress and cell death as observed in MEL cells after GDF11 treatment. Interpretation is further complicated by the observation that GATA‐1 protein can regulate its own expression during erythroid differentiation.^45^

Our results implicate TIF1γ as a likely mechanistic link between Smad2/3 signalling and nuclear GATA‐1 levels. TIF1γ is essential for terminal erythroid differentiation in human CD34^+^ cells and zebrafish, where it functions in a cell‐autonomous manner to regulate transcriptional elongation of genes such as *GATA‐1*.^25^, ^26^, ^37^ Massagué and co‐workers have demonstrated that TGFβ1‐induced formation of pSmad2/3‐Smad4 complexes in haematopoietic stem cells inhibits nuclear availability of TIF1γ because of the competition between Smad4 and TIF1γ for binding to pSmad2/3.^19^ Our data also demonstrated increased nuclear levels of pSmad3 and Smad4, and reduced TIF1γ levels with GDF11 treatment in MEL cells. Interestingly, both Smad4 and TIF1γ require pSmad2/3 for nuclear translocation. We hypothesized that higher pSmad2/3 levels favor complex formation with Smad4 whereas lower pSmad2/3 levels due to inhibition of one or more Smad2/3‐pathway ligands favor formation of pSmad2/3‐TIF1γ complexes. Consistent with this hypothesis, we found that ligand‐mediated overactivation of Smad2/3 reduces nuclear availability of TIF1γ in MEL cells and that inhibition of the Smad2/3 pathway by luspatercept treatment inhibits this reduction in nuclear TIF1γ levels. The parallels between the Massagué findings and our own also raise the possibility that distinct Smad2/3‐pathway ligands use this TIF1γ‐GATA‐1 mechanism to exert analogous effects at different stages of haematopoiesis and erythropoiesis. This could be a challenging hypothesis to test in vivo, however, given that multiple Smad2/3‐pathway ligands appear to concurrently regulate haematopoiesis/erythropoiesis.

Our results in β‐thalassaemic mice also implicate *Hsf1* (heat shock transcription factor‐1) and target gene signatures for protein quality‐control pathways as potentially important mediators of RAP‐536 effects in erythroid precursor cells. Previous studies have noted the importance of protein quality‐control pathways as an erythrocyte defence mechanism against accumulation of unpaired α‐globin chains in β‐thalassaemia.^50^, ^51^, ^52^ Moreover, free α‐globin chains promote GATA‐1 degradation by sequestering heat shock protein normally available for protection of GATA‐1.^53^ Our findings in β‐thalassaemic mice are consistent with the reduced oxidative stress we observed after luspatercept treatment in MEL cells with Smad2/3‐pathway overactivation and could explain reduced formation of Heinz bodies and reduced accumulation of unpaired α‐globin on RBC membranes that we reported previously in β‐thalassaemic mice.^17^

Collectively, our findings highlight the importance of Smad2/3‐pathway overactivation in impaired erythroid differentiation underlying IE in β‐thalassaemia. By sequestering Smad2/3‐pathway ligands, luspatercept prevents overactivation of this pathway and increases nuclear availability of the master erythroid regulator GATA‐1 in erythroid precursors, likely by favouring nuclear complexes of pSmad2/3‐TIF1γ over pSmad2/3‐Smad4. Up‐regulation of GATA‐1 and its target gene signature, as well as heat shock factor and its protein quality pathways, in turn exert coordinated downstream actions that ameliorate oxidative stress and promote erythroid differentiation. β‐thalassaemia is considered a paradigmatic example of IE^54^; therefore, our present mechanistic findings may have wider applicability to related diseases. It remains to be determined how activity by other components of the TGF‐β superfamily^55^ is integrated with mechanisms implicated here.

## CONFLICT OF INTEREST

PMA, BM, RL, HNR, RSP, RK and RNVSS are employees of Acceleron Pharma and/or have ownership interest in the company. MB is Assistant Professor of Medicine at BIDMC and Harvard Medical School, Director of Bioinformatics at BIDMC, and co‐Director of BIDMC Genomics, Proteomics, Bioinformatics and Systems Biology Center.

## AUTHOR CONTRIBUTIONS

PMA, MB, RSP, RK and RNVSS planned and designed the experiments. PMA, RL, HNR, BM, MB and RNVSS conducted the experiments. PMA, RL, HN R., BM, MB and RNVSS collected and interpreted the data. PMA, MB, RK and RNVSS drafted and revised the manuscript.

## Supporting information

Figure S1

Figure S2

Figure S3

## Data Availability

The data that support the findings of this study are available from the corresponding author upon reasonable request.
