# Supplementary figures and images for: Smad2/3‐pathway ligand trap luspatercept enhances erythroid differentiation in murine β‐thalassaemia by increasing GATA‐1 availability
Source: J Cell Mol Med. 2020 Apr 29;24(11):6162–77. doi: 10.1111/jcmm.15243 (PMC7294138; doi:10.1111/jcmm.15243)

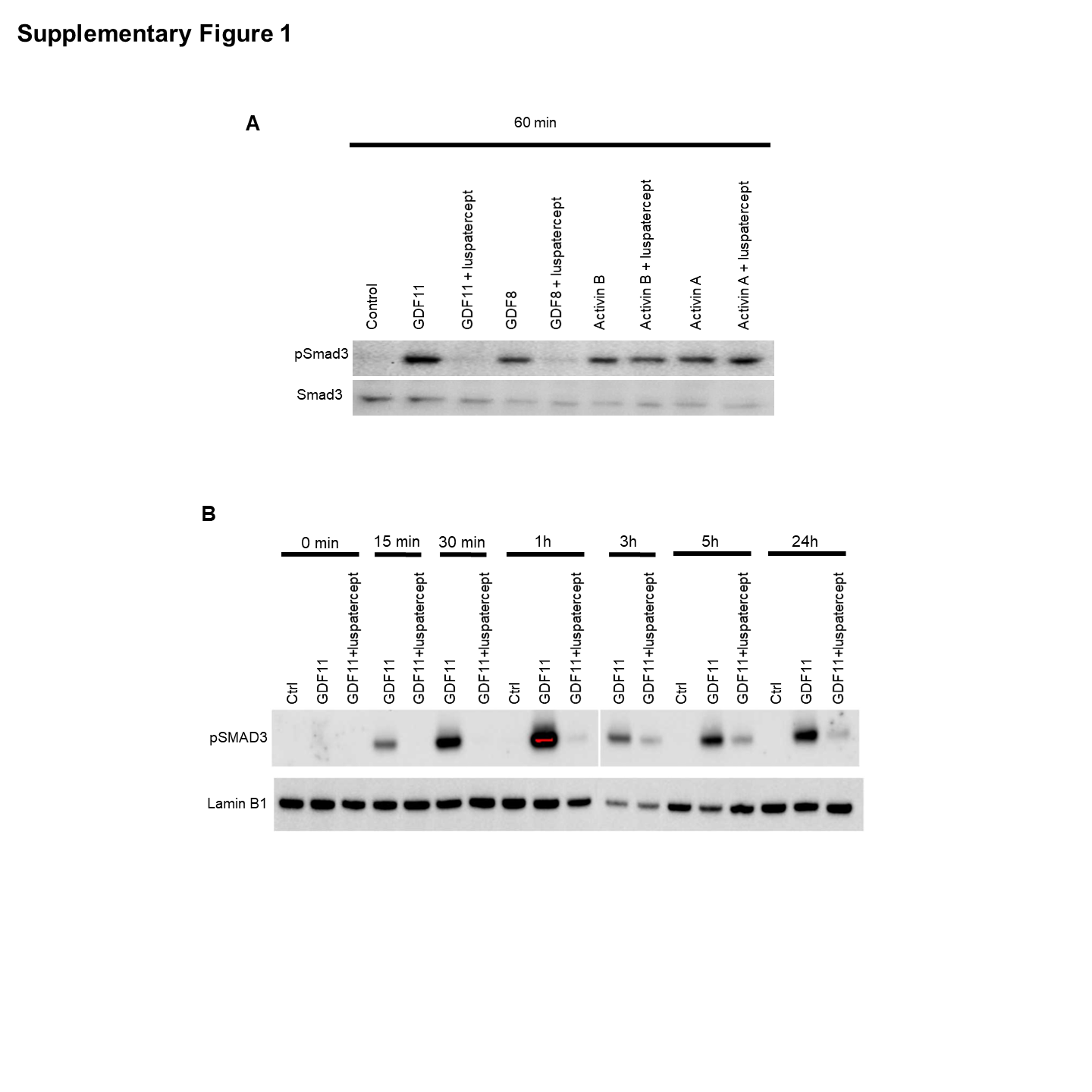

Supplement: Supplementary file 1 — Figure S1 [file JCMM-24-6162-s001.tif]

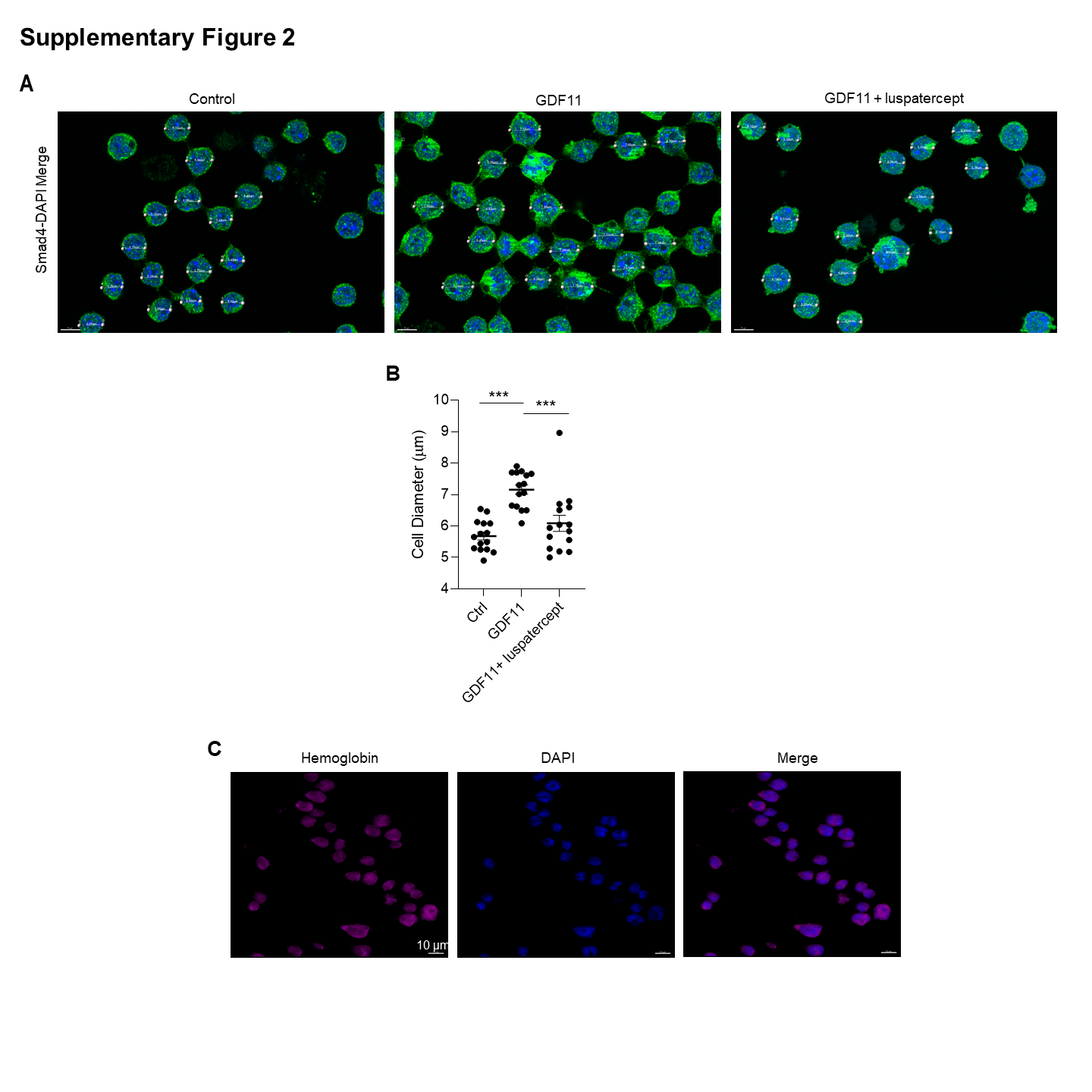

Supplement: Supplementary file 2 — Figure S2 [file JCMM-24-6162-s002.tif]

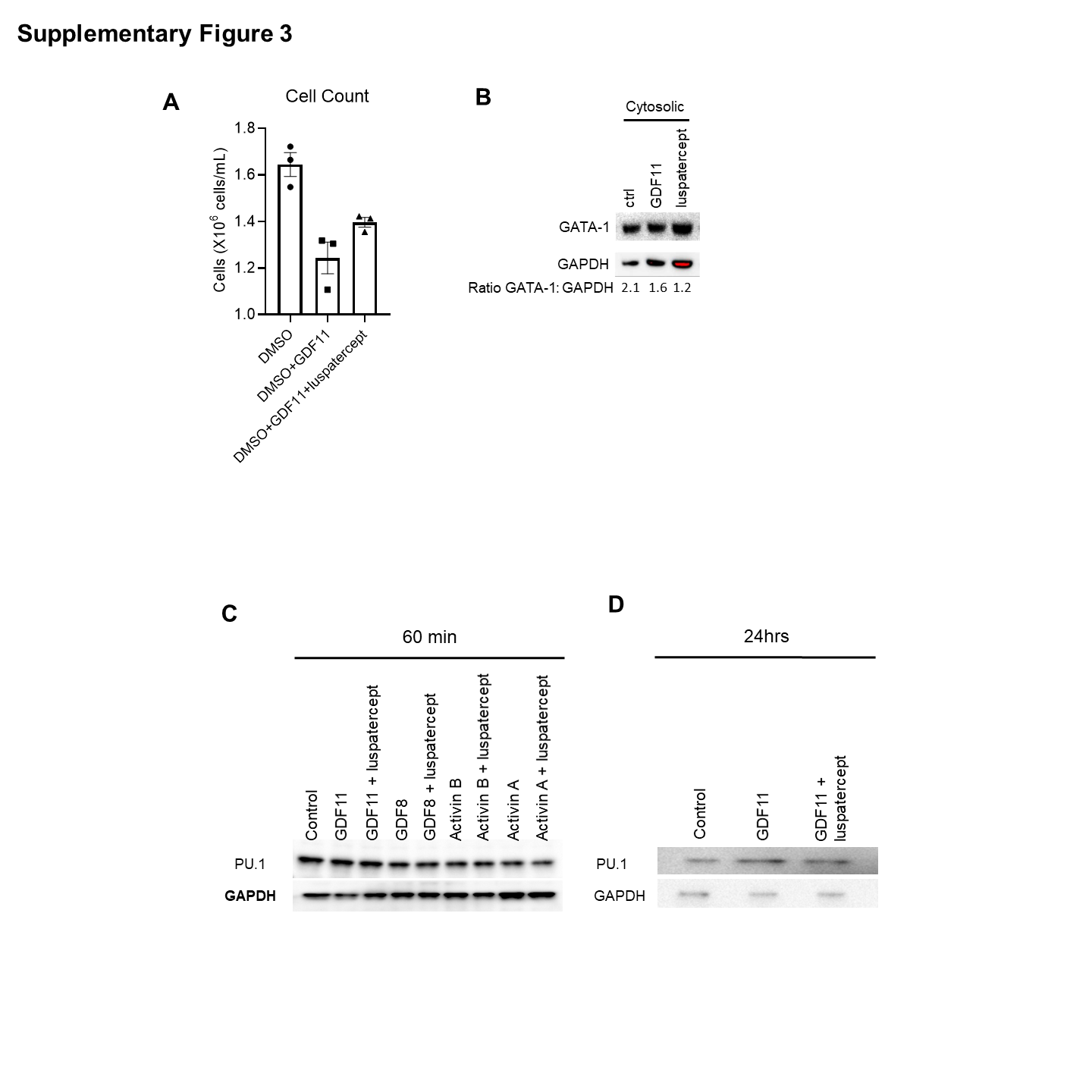

Supplement: Supplementary file 3 — Figure S3 [file JCMM-24-6162-s003.tif]
